# Herausforderungen der Translation von innovativen Produkten und Technologien in die klinische Praxis

**DOI:** 10.1007/s00142-023-00593-3

**Published:** 2023-02-28

**Authors:** Lukas B. Moser, Stefan Nehrer, Peter Angele, Matthias Aurich, Felix Dyrna, Wolfgang Hackl, Silvan Hess, Markus Neubauer, Philipp Niemeyer, Marco-Christopher Rupp, Johannes Zellner, das AGA-Komitee Innovation und Translation

**Affiliations:** 1 Klinische Abteilung für Orthopädie und Traumatologie, Universitätsklinikum Krems, Krems an der Donau, Österreich; 2 Zentrum für Regenerative Medizin, Universität für Weiterbildung Krems, Krems an der Donau, Österreich; 3 Sporthopaedicum, Regensburg, Deutschland; 4 Klinik für Unfall- und Wiederherstellungschirurgie, Universitätsklinikum Regensburg, Regensburg, Deutschland; 5 Universitätsklinikum Halle (Saale), Halle, Deutschland; 6 Gelenkzentrum-Rose, Richard Lehmann-Str. 21, 04275 Leipzig, Deutschland; 7 Universitätsklinik für Orthopädie und Traumatologie, Medizinische Universität Innsbruck, Innsbruck, Österreich; 8 Universitätsklinik für Orthopädische Chirurgie und Traumatologie, Inselspital, Freiburgstrasse, 3010 Bern, Schweiz; 9 OCM – Orthopädische Chirurgie München, Steinerstr. 6, 81369 München, Deutschland; 10 Albert-Ludwigs-University, Freiburg, Deutschland; 11 Sektion Sportorthopädie, Klinikum rechts der Isar, Technische Universität München, Ismaninger Str. 22, 81675 München, Deutschland; 12 Fakultät für Gesundheit und Medizin, Universität für Weiterbildung Krems, Dr.-Karl-Dorrek-Str. 30, 3500 Krems an der Donau, Österreich

**Keywords:** Translationale Medizin, Evidenzbasierte Medizin, Innovation, Minced cartilage, Autologe Chondrozytentransplantation, Translational medicine, Evidence-based medicine, Innovation, Minced cartilage, Autologous chondrocyte transplantation

## Abstract

In Zeiten der zunehmenden Technisierung und Digitalisierung hat die Bedeutung der translationalen Medizin zugenommen. Eine erfolgreiche Translation von der Grundlagenforschung bis zur klinischen Anwendung ist ein kostspieliger und zeitaufwendiger Prozess, der von vielen Faktoren abhängt. Negative Beispiele aus der Vergangenheit (Thalidomid, Metall-Metall-Paarungen bei der Hüftendoprothetik) zeigen, dass eine Translation auch Risiken für die Patienten birgt. In den letzten Jahren wurden strengere Auflagen für die Herstellung und Zulassung von Medizinprodukten eingeführt, um die Patientensicherheit gewährleisten zu können. Die autologe Chondrozytentransplantation (ACT) ist ein Beispiel für eine gelungene Translation. Auf präklinische experimentelle Tierstudien folgten klinische Patientenstudien mit einer Marktzulassung und Implementation in den klinischen Alltag. Die Wirksamkeit allein ist nicht entscheidend, ob dieses Produkt auf dem Markt zugelassen wird. Zwischen der Grundlagenwissenschaft und der Bereitschaft des Markts, in die Weiterentwicklung und Kommerzialisierung eines Produkts zu investieren, ist eine erhebliche Hürde, die auch *Tal des Todes* genannt wird. Nur wenn diese Hürde überwunden wird, kommt es letztendlich zur Marktzulassung und zum klinischen Einsatz. Das Minced-cartilage-Verfahren zur Behandlung von fokalen Knorpelschäden hat kürzlich diese Hürde genommen und den Translationsprozess abgeschlossen. Aktuell liegen lediglich Kurzzeitergebnisse vor; frühzeitige Anwender verwenden diese Technik bereits, obwohl noch keine randomisierten Studien und mittelfristige Ergebnisse vorliegen. Weitere Studien werden zeigen, ob sich ein klinischer Nutzen abzeichnet und das Produkt am Markt bleibt.

## Abstract (en)

In times of increasing technicization and digitalization, the importance of translational medicine has increased. A successful translation from basic research to the clinical application is a costly and time-consuming process that depends on many factors. Negative examples from the past (thalidomide, metal-on-metal bearings in hip arthroplasty) show that translation also entails risks for the patient. In recent years, stricter requirements for the manufacture and approval of medical devices have been introduced to ensure patient safety. Autologous chondrocyte transplantation (ACT) is an example of successful translation. Preclinical experimental animal studies were followed by clinical patient studies with market approval and implementation in the routine clinical practice. The effectiveness of a product is not the only decisive factor in whether a product is approved for the market. Between the basic science and the willingness of the market to invest in the further development and commercialization of a product is a significant hurdle, which is also called the *Valley of Death*. The product will ultimately be approved for the market and used clinically only if this hurdle is overcome. The minced cartilage procedure for the treatment of focal cartilage defects recently passed this hurdle and completed the translation process. Only short-term results are currently available and early users are already using this technique although randomized studies and medium-term results are not yet available. Further studies will show whether a clinical benefit emerges and the product stays on the market.

Das ultimative Ziel der Medizin ist es, den PatientInnen die bestmögliche Therapie zukommen zu lassen. In den letzten Jahrhunderten versuchte man, dies zu erreichen, indem man aus Einzelerfahrung lernte. Dieser eminenzbasierte Zugang wurde in jüngster Zeit von der evidenzbasierten Medizin (EbM) abgelöst – die medizinische Behandlung soll auf Grundlage von empirisch nachgewiesener Wirksamkeit getroffen werden [1]. Die Einführung der Evidenzgrade standardisierte und objektivierte die wissenschaftliche Aussagefähigkeit. In Zeiten der zunehmenden Technisierung und Digitalisierung vernetzt sich die Medizin immer mehr mit anderen Berufsgruppen. Sei es in der Pharmatherapie, Biotechnologie, Materialwissenschaft und vielen anderen Disziplinen – die Zeiten, in denen Ärzte allein Therapiekonzepte entwickeln, sind vorbei. In den letzten Jahren wurde der Begriff der *translationalen Medizin* eingeführt und vielseitig beworben, wobei eine eindeutige Definition bisher fehlt. Das vielzitierte Konzept der translationalen Forschung „from bench to bedside“, also vom Labor zum Patientenbett, greift etwas zu kurz. Prinzipiell geht es darum, Ergebnisse der Laborarbeit in den klinischen Alltag einzuführen. Dies gelingt über präklinische Studien und die Erprobung der Wirksamkeit in klinischen Studien. Neue Produkte sollen effektiv und sicher in den klinischen Alltag translatiert werden. Eine erfolgreiche Translation erfordert ein interdisziplinäres Team (je nach Forschungsgebiet) mit einem vielfältigen beruflichen Hintergrund: Biologie, Biotechnologie, Chemie, Physik, technische Berufe, Projektmanager, Anwälte, IT-Experten etc. [2]. Eine erfolgreiche Translation kann nur gelingen, wenn Ärzte Teil dieses Prozesses sind, da sich Fragestellungen meist aus dem klinischen Alltag ergeben. Ziel der Forschung ist es, zunächst diese Fragestellungen im Labor beantworten zu können. Zusätzlich sind Ärzte nicht nur an der Formulierung der Forschungsfrage beteiligt, sondern auch an der Vollendung der translationalen Medizin – nämlich der klinischen Anwendung der Forschung am Patienten. Dementsprechend ist die translationale Medizin eigentlich nicht als *Einbahnstraße* zu sehen, sprich: vom Labor zum Patienten. Vielmehr ist es ein Kreislauf vom Patienten ins Labor und wieder zum Patienten, in dem ÄrztInnen eine tragende Rolle spielen. Selbst in der Produktentwicklung sind ÄrztInnen als Medical Advisors tätig und damit eigentlich an jedem entscheidenden Schritt der Translation beteiligt.

Das steigende Interesse an der translationalen Medizin spiegelt sich auch in der Wissenschaft wider. Eine PubMed-Suche (27.10.2022) ergibt bei dem Suchbegriff „Translationale Medicine“ 55.177 Treffer. Allein 2021 wurden 11.029 Publikationen verzeichnet, 10 Jahre zuvor (2011) waren es gerade einmal 552. Zusätzlich etablieren sich Journale, welche sich ausschließlich auf die translationale Medizin spezialisieren (z. B. *Journal of Translational Medicine, Science Translational Medicine, Clinical and Translational Medicine, Journal of Orthopaedic Translation* und einige mehr). Ein Großteil dieser Journale ist in PubMed gelistet und ein über die Jahre steigender Impact Factor zeichnet sich ab. Außerdem bieten einige Universitäten Studiengänge an, die eine Vertiefung in die translationale Medizin anbieten (z. B. Translational Medicine Masters am University College in London).

## Beispiele fehlgeschlagener Translation

Die Bedeutung einer erfolgreichen Translation zeigen negative Erfahrungen aus der Vergangenheit. Thalidomid wurde in den 1950er Jahren in Westdeutschland ursprünglich als Schlaf- und Beruhigungsmittel entwickelt. Da sich auch eine Wirksamkeit gegen die morgendliche Schwangerschaftsübelkeit zeigte, wurde Thalidomid für schwangere Frauen empfohlen und beworben. Zu dieser Zeit wusste man nicht, dass dieses Medikament Geburtsdefekte (z. B. Phokomelie, Amelie) verursachen kann [3]. Erst nachdem tausende Fälle beschrieben wurden, wurde das Medikament vom Markt genommen und strengere Richtlinien für die Herstellung und klinische Anwendung von Medizinprodukten eingeführt.

Ein Beispiel aus der Orthopädie ist der klinische Einsatz von Metall-Metall-Paarungen („metal on metal“, MoM) bei Hüftprothesen [4]. Zur Markteinführung der MoM-Paarung in den 1990er Jahren wurden hauptsächlich Metall-Kunststoff-Paarungen verwendet. In dieser Zeit war man mit zunehmend jüngeren Patienten konfrontiert, die körperlich aktiver waren. Infolgedessen waren Dislokationen eine häufige und gefürchtete Komplikation der Metall-Kunststoff-Paarungen. Ein weiteres Problem dieser Paarungen war die Abnutzung nach 10–20 Jahren. Nun erhoffte man sich, über die MoM-Paarungen beide Probleme (Dislokation und Abrieb) lösen zu können. Mechanische Untersuchungen zeigten, dass es bei MoM-Paarungen keinen vergleichbaren Abrieb gab wie bei den Metall-Kunststoff-Paarungen. Zusätzlich zeigten weitere Studien, dass die MoM-Paarung stabil war, mit zudem wenig Dislokationspotenzial. Sie sollte somit eine lange Haltbarkeitsdauer mit einer geringen Dislokationsrate gewährleisten, ideal für das zunehmend jüngere Patientengut. Damals wusste man noch nicht, dass es im Fall einer zu vertikal gerichteten Pfannenorientierung zu einem erhöhten Kantendruck kam. Die Folge dieses erhöhten Kantendrucks war, dass Metallpartikel freigesetzt wurden, welche lokale Entzündungsreaktionen verursachten und in Extremfällen zu einer Schädigung der Muskulatur führten [5]. Viele Patienten erhielten eine Revisionsoperation, und manche erlitten dauerhafte Schäden, da trotz Revisionsoperation keine stabilen Verhältnisse erreicht werden konnten [6].

All diese Beispiele haben gezeigt, dass der Translationsprozess versagen kann, und auch, dass regulatorische Prüforgane (wie z. B. die Food and Drug Administration, FDA) falsche Entscheidungen treffen können. Interessanterweise ereigneten sich diese Beispiele, bevor der Begriff *translationale Medizin* geprägt wurde. Sicherlich hat man aus diversen negativen Erfahrungen gelernt und strengere Auflagen für die Herstellung und Zulassung von Medizinprodukten eingeführt. Dennoch gibt es Sonderfälle, bei denen der Translationsprozess differenziert betrachtet werden muss, vor allem dann, wenn unmittelbarer Patientennutzen mit einem schnellen Handeln gewährleistet werden kann. In Zeiten der COVID-19-Pandemie versuchte man, innerhalb von kürzester Zeit einen wirksamen Impfstoff mit möglichst wenigen Nebenwirkungen herzustellen und für Milliarden Menschen zu produzieren und zur Verfügung zu stellen. Eine große Herausforderung, wenn man bedenkt, dass üblicherweise die Entwicklung eines Impfstoffs etwa 10 Jahre dauert. Nach ungefähr einem Jahr waren mehrere Impfstoffe auf dem Markt. Hier wurde der traditionelle Translationsprozess außer Kraft gesetzt und durch Notfallzulassungen eine schnelle Bereitstellung der Impfstoffe ermöglicht [7]. Ein beschleunigter Translationsprozess erfordert immer eine ausreichende ethische Diskussion, da hierbei potenzieller individueller Schaden (Nebenwirkungen) einem Benefit der Gemeinschaft gegenübersteht.

## Bespiel gelungener Translation

Ein positives Beispiel für eine gelungene Translation ist die autologe Chondrozytentransplantation (ACT) zur Behandlung von fokalen Knorpelschäden. Eine Tierstudie, durchgeführt an Hasen, zeigte 1984 eine erfolgreiche Reimplantation von kultivierten autologen Chondrozyten [8]. Die Chondrozyten wurden unter einem Periostlappen in den Knorpeldefekt eingebracht und der Defekt übernäht. Nach einem Jahr konnte gezeigt werden, dass ungefähr 70 % des Defekts mit knorpelähnlichem Gewebe bedeckt war. Aufbauend auf dieser Tierstudie führten Brittberg et al. eine Landmark-Studie an 23 Patienten mit tiefreichenden Knorpeldefekten des Kniegelenks durch und konnten in den Transplantaten hyalinen Knorpel nachweisen [9]. Drei Jahre später folgte die Zulassung über die FDA in den USA.

Die ACT hat sich zum Goldstandard in der Behandlung fokaler Knorpeldefekte entwickelt

Im Laufe der Zeit kam es über klinische Erfahrungen und Forschungsarbeit zu Weiterentwicklungen dieser sog. „1. Generation ACT“. Um Limitationen wie Hypertrophie oder Ossifikation des Periosts zu verhindern, wurde die „2. Generation ACT“ eingeführt [10]. Statt des Periosts sollen hier eine Kollagenmembran oder synthetische Materialien für eine gleichmäßige Verteilung der Chondrozyten im Defekt sorgen. Zuletzt wurde die „3. Generation ACT“ vorgestellt: Chondrozyten werden in ein dreidimensionales Baugerüst eingebettet. Hier sind weder Nähte noch Periost notwendig, das Gerüst wird mittels Fibrinkleber im Defekt fixiert [11]. Die klinische Fragestellung nach der optimalen Behandlung von fokalen Knorpeldefekten führte nach einer erfolgreichen Tierstudie zu einer ebenfalls erfolgreichen Patientenstudie, welche die Wirksamkeit am Menschen nachwies. Es resultierte eine Marktzulassung und eine Implementation in den klinischen Alltag. Erfahrungen aus Nachuntersuchungen und intensive Forschung in interdisziplinären Teams haben zu einer Verbesserung dieser Technik geführt. Daraufhin hat sich die ACT im Laufe der Jahre zum Goldstandard in der Behandlung fokaler Knorpeldefekte entwickelt [12]. Interessanterweise ist in den letzten Jahren wieder ein Rückgang der Anwendung zu bemerken, wobei hier sowohl ein reduziertes Angebot als auch eine reduzierte Nachfrage eine Rolle spielen. Mittlerweile bieten nur wenige Firmen das Produkt an. Dies liegt an den immer aufwendiger werdenden regulatorischen Hürden und den steigenden Kosten, um diese Kriterien zu erfüllen.

## Herausforderungen der translationalen Medizin

**Figure 1:**
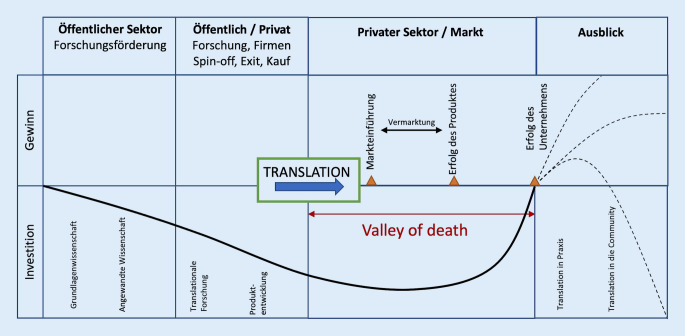

Die translationale Medizin steht vor großen Herausforderungen. Einerseits muss die Translation von der Grundlagenforschung in klinische Studien gelingen (Abb. 1). Andererseits sichern erfolgreiche klinische Studien aber nicht, dass das zugelassene Produkt tatsächlich im klinischen Alltag zur Anwendung kommt. Ein entscheidender Schritt ist die Translation von klinischen Studien in die ärztliche Tätigkeit, welcher von verschiedenen Faktoren abhängig ist. Selbst wenn die Zulassung eines medizinischen Produkts erfolgt, müssen Krankenhausträger und Versicherungen nicht unbedingt einen Einsatz befürworten und entgelten. Die positive Entscheidung hierfür hängt in erster Linie vom Erfolg des jeweiligen Produkts ab, wobei als Erfolg nicht nur der medizinische Nutzen bewertet wird, sondern auch der finanzielle Ertrag. Die Produktentwicklung, ausgehend von der Grundlagenwissenschaft bis zur klinischen Anwendung (ermöglicht durch die Industrie), ist ein sehr kostspieliger und zeitaufwendiger Vorgang. Hierbei kommt es üblicherweise zu einer Entkopplung der Wissenschaft und der Industrie, die als „valley of death“ oder übersetzt als *Tal das Todes* bekannt ist [13]. Dieser Begriff wurde ursprünglich von Ökonomen geprägt, um die Lücke zwischen der Grundlagenwissenschaft und der Bereitschaft des Markts, in die Weiterentwicklung und Kommerzialisierung eines Produkts zu investieren, zu beschreiben [14]. Diese Lücke kann verschiedene Ursachen haben: technische Risiken, Unsicherheit der Finanzmärkte sowie die Wahrscheinlichkeit, tatsächlich einen entsprechenden Ertrag der Investition zurückzubekommen. Um diese Lücke überwinden zu können, sind bedeutende finanzielle Investitionen aus dem privaten Sektor notwendig.

**Figure 2:**
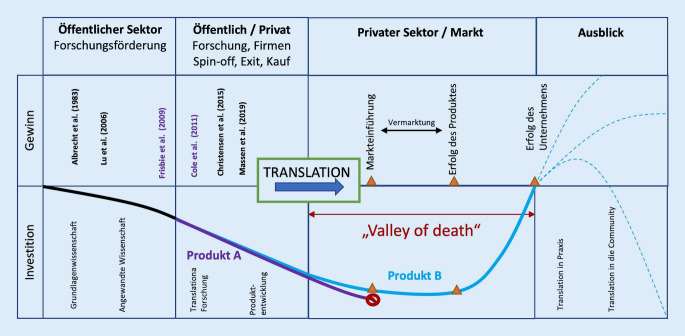

Gelingt dies nicht, scheitert die Translation in die klinische Praxis (Abb. 2).

## Translationsprozess von Minced-cartilage-Verfahren

Dieser langatmige Prozess von der Grundlagenwissenschaft bis in die Praxis kann am Beispiel der Minced-cartilage-Prozedur zur Behandlung von fokalen Knorpelschäden veranschaulicht werden (Tab. 1). In einer Operation wird autologes Knorpelgewebe aus den Randbezirken der Knorpelläsion sowie aus weiteren nichtbelasteten Regionen des Kniegelenks entnommen und im selben Eingriff in den Defekt eingefügt. Das Prinzip, chondrale Läsionen mit Knorpelfragmenten aufzufüllen, wurde bereits 1983 von Albrecht et al. in einem Tiermodell beschrieben [15]. Einige Jahre vergingen ohne neue Studien, bis Lu et al. 2006 in einer weiteren Tierstudie die Bildung von hyalinem Knorpel in Läsionen nach Einbringen von Knorpelfragmenten auf einem bioresorbierbaren Träger beschrieben [16]. Weitere vielversprechende klinische Studien wurden 2015 von Christensen et al. [17] sowie 2019 von Massen et al. [18] veröffentlicht. Zeitgleich wurde im privaten Sektor von einigen Firmen in die Produktentwicklung intensiviert. So wurde die Entnahmetechnik des Knorpelgewebes mittels Shaver-Aufsätzen standardisiert und neue Produkte für die Weiterverarbeitung der Knorpelfragmente entwickelt (z. B. Sammlung von autologem Gewebe mittels Gewebekollektor). Zusätzlich werden eigens entwickelte Produkte für die Bereitstellung von Wachstumsfaktoren aus Blutprodukten sowie die Herstellung einer Matrix aus autologem Thrombinserum beworben. Während eine Firma an dem Translationsprozess scheiterte (Produkt A), konnte eine andere Firma zuletzt eine Markteinführung erreichen (Produkt B). Infolgedessen wird dieses Produkt nun verstärkt auf Kongressen und Fortbildungen beworben, um dessen Erfolg zu sichern. Einige Anwender setzen das Produkt bereits für die Behandlung von fokalen Knorpelschäden ein.

Anfangs leistet eine geringe Anzahl an Anwendern Pionierarbeit und beschreibt ein „proof of concept“

**Figure 3:**
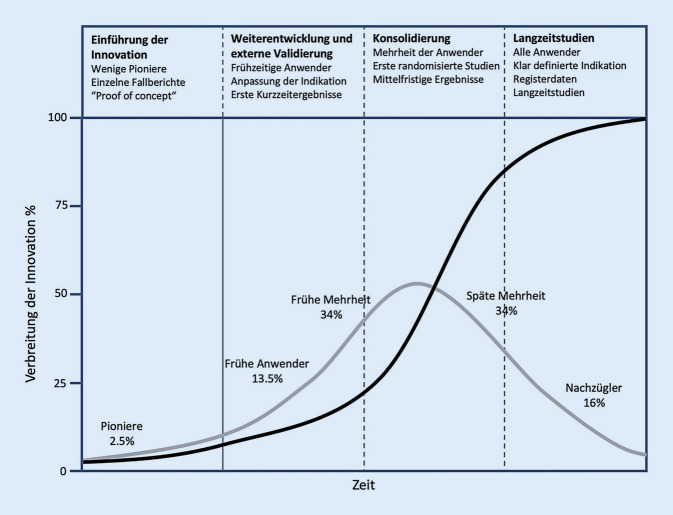

Interessant ist in diesem Zusammenhang die Verbreitung der Innovation innerhalb der Anwender nach erfolgreicher Translation in die Klinik (Abb. 3). Eine geringe Anzahl an Anwendern leistet Pionierarbeit und beschreibt in einzelnen Fallberichten ein „proof of concept“. Es kommt zu einer Weiterentwicklung, wobei frühzeitige Anwender den Pionieren folgen und erste Kurzzeitergebnisse veröffentlicht werden. Erste randomisierte Studien bewirken eine Konsolidierung, und schließlich entscheidet sich die Mehrheit der Anwender für die Anwendung der Innovation. Ein kleiner Teil an Nachzüglern entscheidet sich erst spät, als Langzeitergebnisse und Registerdaten vorliegen. Voraussetzung für diesen Verlauf ist selbstverständlich, dass sich die Innovation als erfolgreich erweist. In Bezug auf Minced-cartilage-Verfahren befinden wir uns momentan in der Phase der Weiterentwicklung und externen Validierung. Aktuell liegen Kurzzeitergebnisse vor, und frühzeitige Anwender verwenden diese Technik bereits, obwohl noch keine randomisierten Studien und mittelfristige Ergebnisse vorliegen. Weitere Studien werden zeigen, ob sich ein klinischer Nutzen abzeichnet und das Produkt am Markt bleibt.

**Table 1:** 

T0	T1	T2	T3	T4
Grundlagenforschung	Translation zum Menschen	Translation zum Patienten	Translation in die Praxis	Translation in die Community
Präklinische Studien Tierstudien	„Proof of concept“ Klinische Studien Phase 1	Klinische Studien Phase 2 Klinische Studien Phase 3	Klinische Studien Phase 4 Clinical Outcome Research	Population-level outcome research
Definition des Wirkmechanismus und des Ziels	Neue Behandlungsmethode	Kontrollierte Studien zeigen effektive Wirkung	Lieferung der zielgerichteten Therapie zum richtigen Patienten	Wahre Nutzen für die Gesellschaft
Albrecht et al. (1983) [15] Lu et al. (2006) [16] Frisbie et al. (2009) [19]	Christensen et al. (2015) [17] Massen et al. (2019) [18]	Cole et al. (2011) [20]	–	–
Translation von der Grundlagenforschung zum Patienten			Translation neuer Daten in die Klinik	

Translationsschritte am Beispiel von Minced-cartilage-Verfahren. Nach durchgeführten präklinischen Tierstudien (Albrecht et al., Lu et al., Frisbie et al.) erfolgte die Translation der neuen Behandlungsmethode zum Patienten (Christensen et al., Massen et al.). Es folgte die Translation am Patienten mit einer randomisiert kontrollierten Studie (Cole et al.)

Gerade in diesem Grenzbereich (Produkte sind auf dem Markt gekommen und es gibt noch keine entsprechenden klinische Studien, die die Wirksamkeit zeigen), nehmen Fachgesellschaften eine bedeutende Rolle ein. Das Komitee „Innovation und Translation“ hat es sich zur Aufgabe gemacht, den Translationsprozess von neuen Produkten objektiv zu evaluieren, um anschließend nachvollziehbare Empfehlungen für KollegInnen abgeben zu können.

## Fazit für die Praxis

- Die erfolgreiche Translation von innovativen Produkten und Technologien in die klinische Praxis ist ein aufwendiger und kostspieliger Prozess, der von vielen Faktoren abhängig ist.
- Das *Tal des Todes* beschreibt die Lücke zwischen der Grundlagenwissenschaft und der Bereitschaft des Markts, in die Weiterentwicklung und Kommerzialisierung eines Produkts zu investieren. Nur wenn diese Hürde überwunden wird, kommt es zum klinischen Einsatz.
- Die autologe Chondrozytentransplantation (ACT) ist ein Beispiel für eine gelungene Translation.
- Das Minced-cartilage-Verfahren zur Behandlung fokaler Knorpelschäden hat kürzlich den Translationsprozess abgeschlossen. Aktuell liegen Kurzzeitergebnisse vor, und die Technik wird bereits von einigen Anwendern verwendet.
- Randomisierte Studien und mittelfristige Ergebnisse gibt es noch keine.
- Mittelfristige sowie Langzeitergebnisse werden über den zukünftigen Erfolg dieses Verfahrens entscheiden.
